# The introduction and implementation of open dialogue in a day center in Athens, Greece: experiences and reflections of mental health professionals

**DOI:** 10.3389/fpsyg.2023.1074203

**Published:** 2023-05-25

**Authors:** Marina Christina Skourteli, Philia Issari, Lito Dimou, Ariadni O. A. Antonopoulou, Georgia Bairami, Artemis Stefanidou, V. Kouroglou, Stelios Stylianidis

**Affiliations:** ^1^E.P.A.P.S.Y.—Association for Regional Development and Mental Health, Athens, Greece; ^2^Laboratory for Qualitative Research in Psychology and Psychosocial Well-being, National and Kapodistrian University of Athens, Athens, Greece; ^3^Laboratory of Psychopathology, Social Psychiatry and Developmental Psychology, Panteion University, Athens, Greece

**Keywords:** open dialogue, implementation, mental health multidisciplinary team, action research, interpersonal dynamics

## Abstract

**Introduction:**

The present study is part of a large-scale original action-research project aiming to assess the introduction and implementation of the Open Dialogue approach within the clinical practice of an established multidisciplinary team in a Day Centre in Athens, Greece. More specifically, it aimed to explore the experiences of professionals within the process of implementation both in relation to their clinical practice and their professional identity.

**Methods:**

Data collection employed a focus group, which was set up to explore professional reflections of the implementation and research processes since the introduction of the model. Thematic Analysis of transcripts revealed two main themes that correspond to the impact of Open Dialogue on professionals’ clinical practice and on team dynamics, respectively.

**Results:**

Professionals identify several challenges in implementing OD, such as difficulties in linking theory to practice, containing uncertainty, and addressing cultural barriers to dialogical ways of working. Professionals further reflect on their own internal journey stemming from the implementation of Open Dialogue that has led them to greater openness and growth, personally and as a team.

**Discussion:**

The role of mental health professionals is being acknowledged as being at the frontline of any meaningful psychiatric reform through the assimilation and promotion of humanistic paradigms aiming towards a change of culture in psychiatric care across different contexts. Despite variations in implementation across different contexts, the importance of consolidating and embracing Open Dialogue as a philosophical framework underpinning mental health care is being discussed.

## 1. Introduction

The Open Dialogue approach constitutes an alternative to traditional psychiatric care for individuals experiencing mental health difficulties, particularly psychosis, and marks an inherently democratic shift in mental health care by introducing service user social network (including mental health professionals) as an integral element of their recovery and psychosocial rehabilitation. Open Dialogue is distinct from conventional approaches to mental illness in that mental health crises are understood as relational—existing in the relationships between people—as opposed to individualistic—located solely within the individual; equally, the goal of therapy is not to treat disease but to support dialogue within social networks rather than changing the service user’s behavior *per se* (Dawson et al., 2019).

Existing limitations of the biomedical model and the often-ambivalent attitudes of professionals regarding service user rights further highlight the need for a structural reform in psychiatric care aiming at the democratization of mental health care (Stylianidis, 2019a,b; Florence et al., 2020). The Open Dialogue approach re-conceptualizes dominant notions of mental illness and underpins an essential move towards psychiatric reform and service user empowerment that values service user and family member experiences as important knowledge bases (Gordon et al., 2016). In that respect, Open Dialogue is not only a novel psychotherapeutic approach but also proposes a new way of organizing and structuring responsive and coherent mental health services that ensure continuity of care (Buus et al., 2017; Dawson et al., 2019).

The Open Dialogue approach and its role in the prevention of relapse and promotion of mental health has been systematically applied in Scandinavian countries, Northern Europe, Australia and the US with culturally specific modifications in order to adapt to different mental health services and contexts (Buus et al., 2017; Gidugu, 2017; Stockmann et al., 2017; Dawson et al., 2019, 2020; Tribe et al., 2019; Florence et al., 2020). The role of mental health professionals is being acknowledged as increasingly vital in promoting the psychosocial integration of service users and in challenging dominant psychiatric paradigms (Buus et al., 2022). In that respect, mental health professionals are at the frontline of a meaningful implementation of Open Dialogue through the assimilation and promotion of democratic, humanistic principles aiming towards a change of culture in psychiatric care across different contexts.

### 1.1. Implementation of open dialogue across different cultures

Most studies on OD implementation attempts have taken place in Scandinavian countries (Buus et al., 2017), with few qualitative studies focusing on the experiences of mental health professionals in introducing or implementing Open Dialogue in their clinical work, across other cultural contexts (Dawson et al., 2020).

#### 1.1.1. Implementation of OD in Scandinavian and Nordic countries

Buus et al. (2017) undertook a scoping review of OD implementation studies across Scandinavian countries. Thylstrup (2009) reports that whilst service users ascribed much value to relationships and in transcending social isolation as a result of Open Dialogue interventions, staff found it challenging to collaborate with professionals from other disciplines, and often felt inadequate in providing Open Dialogue. Similarly, Johansen and Bille (2005), report that the purpose and aims of network meetings were not always clear to network members, nor was the professionals’ level and type of engagement primarily due to the cautious attitude of professionals towards the approach. The authors suggest that the Open Dialogue approach ought to be used in families whose thinking is somewhat aligned with such an unconventional approach to mental health, thus posing the issue of therapeutic match between approach and client. Sjømæling (2012) further reports that professionals felt that network meetings were personally challenging because of high levels of uncertainty and disclosure. Such professional uncertainty with regard to the level and type of involvement is also reported by Piippo and Aaltonen (2008), who found that participants who had received Open Dialogue interventions described mistrust in situations where the professionals’ team was experienced as either over-involved or uncertain and ambivalent in taking decisions. Similar research reports that whilst mental health professionals overall seem to evaluate the Open Dialogue positively in enhancing their clinical skills and attitude, they nevertheless struggle with abandoning their usual expert role and with maintaining a not-knowing stance towards the outcome of dialogical position (Brottveit, 2002; Bjørnstad, 2012; Schubert et al., 2020).

Johansen and Weber (2007) report resistance towards the implementation of OD at an individual, organizational, and professional level. Clinicians in their study found it challenging to refute their expert role and establish a new type of expertise that would both accommodate the non-hierarchical structure of the approach as well as maintain their professional identity. Similarly, Sondergaard (2009) reports that despite attempts to implement the Open Dialogue approach in a small outreach mental health team in Denmark, professionals eventually abandoned the project during the process of its implementation. Holmesland et al. (2010) and Holmesland et al. (2014) also explored the experiences of healthcare professionals working in a dialogical way. Findings revealed that professionals were able to develop a trans-professional identity and role, however the greatest challenge was to foster the professionals’ ability to genuinely listen. Interestingly, less experienced professionals without formal therapeutic training were reported as being better able to integrate Open Dialogue skills into their practices, a finding also reported by Clement and McKenny (2019).

Overall, findings from Nordic and Scandinavian countries suggest that the introduction of Open Dialogue often generated resistance from practitioners, whose position and identity were challenged in several ways; in some cases, findings implied a lack of genuine engagement and understanding of dialogism by professionals. Finally, reports highlighted that not everyone experienced Open Dialogue positively. For example, families with a strong belief in authority and an expectation of being directed by mental health professionals may find the open format of the approach confusing and frustrating. The small body of research examining Open Dialogue implementation in Scandinavia suggests that the adoption of the Open Dialogue principles require significant organizational change, which may in turn generate organizational, professional and personal resistance (Buus et al., 2017).

#### 1.1.2. Implementation of OD across other cultural contexts

There is very little research from non-Scandinavian countries regarding the introduction of Open Dialogue and no extensive reviews on implementation and organizational processes (Dawson et al., 2019, 2020; Freeman et al., 2019; Florence et al., 2020).

In a couple of Australian studies, Dawson et al. (2019, 2020) report that despite professionals’ openness and supportive attitude towards the approach, existing organizational ideology and structures clashed with the integration of Open Dialogue principles. Dialogical ways of working were challenged by the dominant medical model and the emphasis placed upon economic efficiencies by the organization. These studies highlight the importance of a ‘good’ fit between organizational culture and efforts to implement recovery-oriented care (Dawson et al., 2019, 2020). In Canada, Florence et al. (2020) further report that even though Open Dialogue is an approach that challenges power differentials in mental health, power dynamics, issues of authority, status and expertise remained prominent within the professionals’ team even after the introduction of the approach. Further, staff reported that whilst giving up power within the treatment setting was positive and liberating, it was somewhat disorienting when it came to issues of risk and suicidality of service users and to re-negotiating aspects of their professional identity (Florence et al., 2020; Schubert et al., 2020). Equally, research on attempts at implementation of Open Dialogue in the United States and the United Kingdom reveals that although Open Dialogue is acknowledged as clinically helpful, training costs and the need to translate OD principles into the local context may constitute barriers to effective implementation (Gordon et al., 2016; Rosen and Stoklosa, 2016; Tribe et al., 2019; Kinane et al., 2022).

#### 1.1.3. Implementation of open dialogue and organizational change

Taken together, implementation studies suggest that the adoption of Open Dialogue requires significant organizational change. Research on implementation attempts outside Scandinavian countries, further highlight the importance of context and culture and the ways in which such parameters may affect effective and long-term implementation. Still, the paucity of research across different cultural contexts limits our understanding of the perceived benefits and challenges to fully implementing OD-informed approaches successfully (Dawson et al., 2019, 2020; Freeman et al., 2019; Florence et al., 2020). The relative success or failure of any implementation may be attributed to diverse social, cultural and organizational factors including the broader social, economic, cultural and political contexts (Damschroder et al., 2009; Dawson et al., 2019, 2020). The available research emphasizes the need for careful organizational consideration and commitment in order to ensure that the professionals involved both understand Open Dialogue and find it an acceptable and realistic socio-cultural fit to local conditions (Gidugu, 2017; Dawson et al., 2019; Ong et al., 2019; Tribe et al., 2019).

Variation in models of Open Dialogue across different settings, heterogeneity of methodologies following the implementation process and lack of consistency in implementation strategies mean that thorough descriptions of implementation are still lacking in the literature and that more research is needed to support implementation efforts as well as organizational and professional adjustment to dialogical ways of working (Freeman et al., 2019; Twamley et al., 2021). Organizational change transcends through different stages and impacts employee values and dynamics (Aarons et al., 2011; Hussain et al., 2018), whilst the outcome of any reform is mediated by professional attitudes towards change, anticipated gains and the quality of the management in containing tension. It is particularly helpful for facilitators of change to maintain ongoing communication and transparency among everyone involved, in order to disseminate information, reduce team anxiety and promote a sense of inclusion as well as psychological and practical commitment (Herscovitch and Meyer, 2002; Weiner et al., 2008; Tribe et al., 2019).

### 1.2. The role of mental health professionals

Research suggests that overall, the OD approach is being welcomed by professionals as a good and inspiring alternative to conventional mental health practices; Open Dialogue seems to be appreciated by mental health professionals, as it socializes them into a dialogical and reflective way of being with the other, characterized by understanding and a willingness to share aspects of oneself (Holmesland et al., 2010, 2014; Buus et al., 2017, Galbusera and Kyselo, 2019; Kinane et al., 2022).

Drawing from Mikhail Bakhtin’s views on dialogism and polyphony (Bakhtin, 1986; Anastasiades and Issari, 2014), the Open Dialogue approach essentially challenges mental health professionals to adopt dialogue and polyphony as the primary vehicle for constructing meaning and change in their clinical practice (Seikkula and Olson, 2003; Stockmann et al., 2017; Buus et al., 2022). Mental health professionals are asked to participate in the dialogue not from a traditional ‘expert’ stance but through their authentic thoughts and feelings; in that respect, they need to be engaged into active listening, promoting space for whatever emerges from the dialogue, without censoring it (Hendy and Pearson, 2020). The challenges that have been identified around the implementation and practice of Open Dialogue, indeed seem to refer to mental health professionals’ difficulties in abandoning traditional professional roles, organizational difficulties in supporting implementation attempts as well as the uncertainty around applying such a relational stance into clinical practice (Buus et al., 2017; Ong and Buus, 2021; Kinane et al., 2022).

In that context, mental health professionals from different disciplines need to challenge their own assumptions around hierarchy and to work towards the cultivation of a democratic culture within the organization (Seikkula and Olson, 2003; Holmesland et al., 2010). Therapist experience and specialization in a specific discipline may indeed be challenging for mental health professionals that are members of a multidisciplinary team as they may actively aim for targeted interventions or solutions perhaps as a means of regulating their own anxiety and need to control therapeutic outcome (Borchers, 2014; Buus et al., 2017; Stockmann et al., 2017; Schubert et al., 2020). Mental health professionals may face challenges in integrating practices that are not taught but rather experientially acquired and require the adoption of a new modus operandi where transparency and acting from a non-expert stance are elementary; further research seems to confirm that Open Dialogue principles may often cause insecurity in mental health professionals that may lead to reduced participation and questioning of the model (Buus et al., 2017; Dawson et al., 2019, 2020; Florence et al., 2020; von Peter et al., 2023).

In this study we will focus on the case of Greece and on the attempts to introduce and implement Open Dialogue within an established mental health service.

### 1.3. Open dialogue in a day care centre in Greece

The present action-research was implemented longitudinally since September 2018, in collaboration with Panteion University (Laboratory of Psychopathology, Social Psychiatry and Developmental Psychology) and National and Kapodistrian University of Athens (Laboratory for Qualitative Research in Psychology and Psychosocial Well-being). The study aimed towards an in-depth understanding of the impact of the introduction of Open Dialogue in a multidisciplinary team of mental health professionals in a Day Centre for Psychosocial Rehabilitation in Athens.

More specifically, *the setting* is a Day Centre for Psychosocial Rehabilitation, a community mental health unit for adults suffering from serious mental health disorders and their families. The multidisciplinary *team* consists of psychiatrists, psychologists, social workers, occupational therapists and psychiatric nurses. Professionals had not attended any certified training in Open Dialogue except for brief introductory seminars delivered online, by Scandinavian colleagues, who had a long experience in the implementation and practice of Open Dialogue. Further, participants were acquainted with Open Dialogue experientially, through the establishment of a weekly Open Dialogue discussion group, a forum created by professionals themselves that aimed at the familiarization, self-education and self-reflection on Open Dialogue practices and any other issues and dynamics that emerged as a result of implementation attempts (Hopper et al., 2019).

The introduction and implementation of the Open Dialogue in the Day Centre has developed over the course of 5 years and can be conceptualized in two phases namely, an earlier phase and a later phase. The aim of the present paper is to present the later phase of the study which focuses on the experiences of professionals within the process of implementation both in relation to their clinical practice and their professional identity. However, as this is a five-year long project, which represents an ongoing, internal process from the part of professionals in relation to Open Dialogue, it seems important to provide a brief summary of the earlier phase of the study in order to depict the development of the journey.

The early phase extended from September 2018 to January 2020. During the early phase two distinct main themes were identified that correspond to two separate time periods with regard to the early phase of the study. Taken together, main themes and subthemes create a coherent story about the team’s journey with Open Dialogue over time (Skourteli et al., 2019, 2021).

During the “*Introductory-Exploratory’ period* the multidisciplinary team felt that was in a position of passivity and disempowerment regarding the implementation of the Open Dialogue approach. The research itself was viewed as part of a vertical hierarchy that imposed the new approach; group dynamics were affected, and initial stages of the introduction were marked by anxiety and suspicion around issues of authority and power. Ambivalence towards the new model was initially expressed through a depreciation of the approach as introducing “nothing new” to treatment as usual (Sondergaard, 2009; Holmesland et al., 2014). The team initially attempted to manage the introduction of the Open Dialogue approach by equating and assimilating it to already existing representations and practices by actively seeking points of convergence between established and novel approaches. Although attractive, the democratizing and deeply reforming nature of Open Dialogue appeared to evoke insecurities with professionals feeling unprepared to engage with it (Skourteli et al., 2019; Stylianidis, 2019b; Schubert et al., 2020). These initial findings seem consistent with literature highlighting the resistance of mental health professional teams in assimilating Open Dialogue as part of their professional practice (Sondergraard, 2009; Thylstrup, 2009; Holmesland et al., 2010, 2014; Seikkula, 2011; von Peter et al., 2023).

Over time, during the ‘*Introductory Systematizing’* period, following significant structural and systemic changes within the service—along with the researchers’ sharing of preliminary findings with the OD team—mental health professionals seemed to gradually move from a position of passivity to one of responsibility and agency with respect to the introduction of the Open Dialogue approach. Monthly team supervision, introduced as part of the research protocol significantly facilitated the necessary space for reflection and supported the Open Dialogue team in becoming more defined. Over time, the Open Dialogue team was able to better integrate dialogical ways of being into their identity and practice, whilst maintaining a realistic view of the challenges and ongoing needs (Skourteli et al., 2021). For a more detailed account of earlier phases of the research, see Skourteli et al. (2019, 2021).

The later phase of the research project presented here, focuses on the overall stocktaking, experiences and reflections of professionals on the implementation of Open Dialogue as well as the challenges and main issues that emerged throughout this process.

## 2. Methodology

The overall project employs an action-research methodology following the introduction and implementation of the Open Dialogue approach within a multidisciplinary team of mental health professionals. Action-research seems an appropriate choice of methodology, since it seeks transformative change in the clinical and organizational aspects of the mental health service presented here, through the simultaneous process of taking action (OD implementation) and doing research, linked together by critical reflection. As its goal is oriented towards organizational change, the knowledge produced and actions undertaken inform each other in cyclical ways over the process of the research (Stringer and Genat, 2004; Issari and Polyzou, 2013).

### 2.1. Participants

In the later phase of the study participated 11 professionals (four psychologists, two psychiatrists, two social workers, an occupational therapist and two mental health nurses). None of the participants had attended any formal OD training but were attending monthly external supervision for the past 2 years, with two senior colleagues that had completed the structured 3-year OD training in the United Kingdom Inclusion criteria for therapists included the implementation of the OD approach in their practice.

### 2.2. Data collection

A focus group was set up that consisted of professionals implementing Open Dialogue principles in their clinical practice. The aim of the group was to explore the overall experience of the implementation process within the service as well as to review and reflect upon the professionals’ journey with Open Dialogue. The focus group was facilitated by the senior researcher overlooking the study (MS) and lasted approximately 2.5 h. The facilitator initially introduced broader questions on the impact of implementation before exploring more specific aspects of participants’ experience. Questions aimed at eliciting narratives on the development and implementation of the Open Dialogue approach within the Day Centre. Some examples included: what is your experience of Open Dialogue? how has your experience evolved over time? how has Open Dialogue affected your clinical practice? what are the gains and challenges of implementing this approach? how was your experience of participating in the current research whilst implementing a novel approach? Participants were encouraged to express their experiences and to interact with each other, as the latter prompted new questions that clarified individual and shared perspectives. The focus group was conducted in order to uncover a shared understanding of how aspects of Open Dialogue was implemented and to capture interactions and contrasting perspectives amongst participants (Buus et al., 2022). The focus group was audio-recorded and transcribed verbatim by the senior researcher with indications of basic turn-taking features, including interruptions and overlapping speech (Tong et al., 2007). The quality of the transcripts was assessed by comparing transcriptions to audio recordings, with the help of a second senior researcher, specializing in qualitative research methods, which led to a few corrections of details of the transcripts.

### 2.3. Ethics statement

The present study took place with the informed consent of all participants. The nature and aims of the study were thoroughly explained to members of the multidisciplinary team and written consent was obtained, whilst participants maintained their right to withdraw from the research process until the point of verbatim transcription of the focus group. Collected data were coded to promote anonymity and confidentiality of all participants and were stored electronically in password-protected files only accessible by the researchers; following completion of the research, all data will be permanently destroyed. Finally, participants of the focus group were debriefed about the research process in order to promote transparency and inclusion in the research process (Howitt, 2010; Emerson et al., 2011; Issari and Pourkos, 2015).

### 2.4. Data analysis

Thematic analysis with an experiential and realist orientation (Braun and Clarke, 2006) was utilized for the analysis of data produced from the professionals’ focus group. Audio recordings of the focus group were transcribed verbatim, and transcripts were analyzed inductively in order to reflect the experience of participants. Transcripts were read and re-read by researchers in order to generate some initial codes which were then organized into recurrent patterns or themes in what is being discussed. Produced themes were then reviewed and refined to ensure that themes cohered meaningfully whilst reflecting distinct and identifiable entities that correspond to participant narratives. The researchers followed Braun and Clarke’s (2006) six steps which included familiarization with the data, generation of initial codes, searching for themes, reviewing potential themes, defining and naming them.

## 3. Results

Themes that were produced from thematic analysis of the focus group highlighted the impact that Open Dialogue has had not only upon professional clinical practice, but also on group dynamics and team processes over time. Professionals were able to verbalize clinical concerns and to maintain a critical stance towards the Open Dialogue approach. The participation in the present action-research itself seems to have facilitated team openness and growth both professionally and personally. Overall, two master themes were produced from data analysis with seven corresponding subthemes (three and four subthemes respectively). Table 1 outlines the master themes and subthemes that were produced from the thematic analysis of the professionals’ focus group.

**Table 1:** Master themes and subthemes of professionals’ experiences.

Professionals’ focus group	
Master themes	Subthemes
Impact of implementation of OD on clinical practice	– Difficulties in linking theory with practice of OD
	– Containing uncertainty
	– Cultural fit between OD approach and service user network
Impact of implementation of OD on professionals’ team	– Experience of participating in the research
	– Team openness and growth
	– Challenging team omnipotence and acknowledging own boundaries
	– High turnover of staff

### 3.1. Impact of implementation of OD on clinical practice

The first master theme highlights the impact of the introduction of Open Dialogue upon professionals’ clinical practice. A prominent challenge refers to difficulties linking OD theory and practice, whilst there is an acknowledgement of the experiential aspect of the approach. Professionals are better able to question their stance towards uncertainty and how this may impact ways of being with clients, whilst maintaining a critical stance about the universality of OD and raising the important question of what works for whom in psychotherapy.

#### 3.1.1. Difficulties in linking theory with practice of OD

Professionals expressed their difficulties in bridging the theoretical aspects of Open Dialogue and applying them in their clinical work with clients. This is most likely the outcome of a lack of formal OD training amongst professionals, which may be particularly accentuated as service users’ mental health is often severely affected upon referral. Professionals refer to a sense of ambiguity around ways of being with clients, particularly the notions of therapist reflection and transparency in network meetings.

*‘… It appears to be ideal and captivating when I read about the OD approach in theory, in the literature and through the research process. But when the time comes to apply it in the work with a real person in distress, I think to myself-ok, how can I really apply this, how do I do it? It is not something that can just be applied as a set of skills, this seems to a whole new different context above and beyond myself’ (P4: extract from professionals’ focus group)*

*‘Sometimes I get the sense, what do I do, what I am I trying to do and to what extent do I understand what I am doing. To what extent am I a part of this … Because having read about it is one thing, but having experienced it is quite different … I think I will only be able to do it when I experience it myself. At least this is what I think … I have never in my life been able to learn something just by reading about it. There is a gap there … So I think this is quite difficult’ (P8: extract from professionals’ focus group)*

*‘For me, what still remains quite ambiguous is the part around reflective practice … I am always anxious whether it is appropriate to self-disclose, what is my motive, if the other person should hear it, whether it is helpful I mean for them or whether I would like to share something more private … I think it is a fine balance that can be quite facilitative or meaningful, or on the other hand quite harmful I guess …’ (P1: extract from professionals’ focus group)*

*‘… There is the issue or transparency here, and more precisely even honesty. I can empathize with service user X, I can understand why she is frightened, and I can mirror this-however, when she is telling me about how she is being persecuted by everyone, I cannot confirm this … Perhaps this is something lacking in my training theoretically and practically. Psychotherapy is supposed to be about the reality principle … now you are going to think, which reality? Reality is how the other feels or thinks she feels I guess …’ (P10: extract from professionals’ focus group)*

#### 3.1.2. Containing uncertainty

Professionals are acknowledging the containment of uncertainty and a not-knowing stance as a valuable albeit difficult aspect of the Open Dialogue approach. They are able to reflect on their stance towards knowing and not-knowing stemming from their own anxieties and need to remain in control.

*‘There were times where I felt that my capacity for containing uncertainty was exceeded in relation to the psychotic symptom. It is quite frightening to get into people’s delirium … It was scary to get into this narrative, it was as though we were one and I couldn’t deal with it’ (P7: extract from professionals’ focus group)*

*‘The way I have been trained, you do not get this deep into the symptom, you focus more on reality and you liaise with the healthy part of the person, so to speak … There have been times with my co-therapist where things got quite scary for me, to get used to this and to find my own space and boundaries within all this-I felt like I was losing myself …’ (P7: extract from professionals’ focus group)*

*‘There were times where we had to provide a solution because the meetings were revolving around the same themes, the family was stuck, so we needed a little push, a little problem-solving …’ (P6: extract from professionals’ focus group)*

*‘I think this is about our own issues around working with difficult service users-so I sometimes agree with providing solutions. I think it is related to the severity of the condition as well as our own difficulties with uncertainty, so we resort to more monological interventions-it is safer’ (P3: extract from professionals’ focus group)*

#### 3.1.3. Cultural fit between OD approach and service user network

Participants are maintaining a critical stance towards the universality of Open Dialogue and begin to raise questions regarding the applicability and fit of the approach, both in terms of culture as well as network characteristics and dynamics. In particular, professionals begin to challenge the notion of OD as an ideal therapy and to form more realistic expectations of it. Essentially, the team is reflecting upon the important issue of what works for whom in psychotherapy and raises the issue of how the approach interacts with specific service-user, network and therapist characteristics.

*‘I think the network determines quite a lot of things, as it affects everything else. It all began from the quality of the network and the mentality of each family. Network X was quite easy to work with because they were quite open, network Y was on the other end of the spectrum …’ (P9: extract from professionals’ focus group)*

*‘I saw that not everyone had the patience to see where this is all going to lead … Some people were after a solution now, they wanted to get better. I believe they wanted to carry on with OD but they could not wait for so long, they wanted to feel better now and they underestimated everything else …’ (P2: extract from professionals’ focus group)*

*‘I do not know how to assess this … some families appreciate the small changes stemming from moments in the sessions, others saw nothing helpful at all … I think this is related to the mentality of each family …’ (P4: extract from professionals’ focus group)*

*‘I think the key is to be able to comprehend the other person’s reality and to be able to step in their shoes. Some families cannot do this at all whilst others more so … I think this is an important parameter’ (P5: extract from professionals’ focus group)*

*‘Internal polyphony sometimes is not possible. And it is usually not possible in families where there is emotional unavailability, there is no connection to feelings …’ (P4: extract from professionals’ focus group)*

*‘My thoughts are that OD is not a panacea, it is like all other psychotherapies what works for whom? Like in an individual psychotherapy, you would be able to say when making an assessment that psychoanalysis for example is not a fit with this client. Perhaps it is an approach that doesn’t suit everyone, I don’t know …’ (P1: extract from professionals’ focus group)*

### 3.2. Impact of implementation of OD on professionals’ team

The introduction and implementation of Open Dialogue within an established mental health team seems to have also impacted the dynamics and group processes of the team of professionals over time. The onset of the present action-research and the introduction of the new approach seems to have offered professionals the opportunity to reflect on their own personal, transformative journey over time.

#### 3.2.1. Experience of participating in the research

Professionals are able to reflect upon their experiences of participating in the present action-research and on how this process has evolved over time, especially as Open Dialogue was initially implemented in a top-down manner by the management of the organization. Issues around fears of assessment and anxieties over criticism, although still present to some, seem to have subsided and to have given way to seeing researchers as allies that may operate as organizing and supportive for therapists along the journey of OD.

*‘I never felt that I was being assessed, although the researchers did not speak during network meeting and they were keeping notes, but I never had the feeling of being judged-quite the contrary, what I had in mind is that this person is on our side and she will always have in mind my intention even if I make a mistake …’ (P2: extract from professionals’ focus group)*

*‘At the beginning I was anxious about what they were writing down, the notes they kept, and I could not focus on the session at first but as time moved on, I began to like this, to experience it as a supportive reminder of the Open Dialogue principles and why we were there, and I was more focused …’ (P6: extract from professionals’ focus group)*

*‘I saw her more as a third eye in network meetings, she stood at a greater distance compared to me in relation to the client and she could see more clearly … So, I have always been looking forward to receiving feedback … Having another person that is more external to our team, made me more organized and boundaried, even with scheduling appointments …’ (P5: extract from professionals’ focus group)*

*‘My own feeling was that we were much stricter on ourselves than what we ought to and we expected that somehow from the researchers at the beginning, although this was not the case at all’ (P3: extract from professionals’ focus group)*

*‘I did not have the sense of being assessed, I was just working in the usual way. At the beginning I did not know whether I should speak to her at all but eventually I felt very connected with her, I felt I had someone to lean on, we were chatting on our way back from network meetings and I experienced all this as very helpful …’ (P4: extract from professionals’ focus group)*

#### 3.2.2. Team openness and growth

The theme of the multidisciplinary team’s openness has been ongoing since the onset of the research project and seems to refer to both an external sense of openness and receptivity towards new colleagues and ideas as well as an internal sense of personal growth. It appears that the team has managed to make a significant shift over time towards a stance of greater polyphony and inclusion that is being experienced as enriching and meaningful, personally and professionally.

*‘We became more open as a team, we opened up to more voices, by letting more people in (the researchers), something like what takes place in network meetings amongst ourselves … Like we usually say in systemic therapy, a closed system is the one that perishes in the end, an open system is adaptive and flexible, and I think this is what has happened in our team … Even conflict is not necessarily destructive and doesn’t mean the end …’ (P7: extract from professionals’ focus group)*

*‘I was thinking about openness, not only therapeutically, but here, in our team, how differently we interact we each other. Our morning reflective exercises even in the presence of new people-we were not used to this, and they were not used to us being open and then they became a part of all this. The openness in our team when the researchers came, that was a significant shift’ (P10: extract from professionals’ focus group)*

*‘At the beginning of all this journey we were quite closed as a team I think, it was as though we were into a merger. And anything external, coming from the outside, researchers over the years, new colleagues, we felt as though it was threatening because we also had this Ideal about ourselves that we can manage everything and if we can’t, then we will be judged for it. We thought we were the best because we can manage everything and if we couldn’t then we were the worst. And now, we see that a Third, can enrich us and organize us and we are quite welcoming of this now. I think there has been a great transformation in our team over time, since the introduction of Open Dialogue’ (P1: extract from professionals’ focus group)*

#### 3.2.3. Challenging team omnipotence and acknowledging own boundaries

The introduction of Open Dialogue in a team of experienced mental health professionals, along with the lack of training in the particular approach, seems to have challenged professionals’ sense of expertise, authority and professional identity. Over time, professionals have been able to reflect upon their own professional identities, sense of omnipotence and anxieties over incompetence and criticism (something that may be an outcome of the wider organizational culture), to acknowledge their own limits and to move towards more realistic and meaningful ways of relating to themselves and others.

*‘The longer you work with OD, the more you open up space for your own internal polyphony. And I think being able to hear more aspects of yourself, acknowledging our own limitations and keeping our expectations realistic allows us to say, well this is all that I can do, this is what I can. And I think this is a qualitative change in our team and in every single one of us…’ (P9: extract from professionals’ focus group)*

*‘This year, I saw a change within myself, I do not need to hold people under my wing, I am more ready to acknowledge endings and limits. At some point I did say to my co-therapist, this is enough, we did what we could with this family, which is something I didn’t have before. On one hand, we are no longer after a quick result or an impressive change, we give time and we acknowledge small changes but then there comes a time when time is over, and this is ok …’ (P8: extract from professionals’ focus group)*

*‘We are able to put better boundaries at some point and this older sense that we must have all the answers and solutions otherwise we are bad at our work, we gradually abandon this sense of omnipotence that we are ideal and must be able to manage everything’ (P3: extract from professionals’ focus group)*

#### 3.2.4. High turnover of staff

Participant narratives reflect that the introduction of the Open Dialogue approach is being experienced as having had a significant impact on the organization as a whole and particularly so, amongst the professionals in the Open Dialogue team. There were significant role changes across all levels of the organization, with a number of colleagues departing from the Open Dialogue team either as a result of conflict, promotion to higher management or due to changes in their personal circumstances. For a short period of time, there was a high turnover of staff in the OD team, with several colleagues joining and then leaving the team within a brief period of a few months, something that seems to have caused a sense of discontinuity and instability amongst professionals. Participants are reflecting upon this period and the ways they feel that organizational changes may have impacted their clinical practice.

*‘The first thing that comes to my mind is the departure of colleagues from the team that upset the balance of the therapeutic couples I think and it did cause a discontinuity for a while … A lot of changes took place over time not only in our OD team but also the organization. Many people left, others changed roles and all this on top of the severity of our clients’ mental health can cause a lot of people leaving …’ (P5: extract from professionals’ focus group)*

*‘Since our team changed, with all these departures of colleagues, I got this sense that we will, well and we did, I think, regress to an earlier stage and we were closer to ACT rather than OD. It was around the time when people left, and new people came into the team and I had mentioned it then in our meetings that we became more ACT than OD for a while …’ (P6: extract from professionals’ focus group)*

*‘Well yes, this does make sense, when a system is de-stabilized it is inevitable that it will move towards what is familiar to be able to find its balance again, to find its base before venturing out again and I think the high turnover of colleagues in our team made us, very wisely I think, regress to what we knew best, to maintain our self-esteem until the team is restored and new members are integrated …’ (P3: extract from professionals’ focus group)*

## 4. Discussion

The present study is part of a larger action-research exploring the introduction and implementation of OD within the clinical practice of a multidisciplinary team of mental health professionals. Τhe present study aimed at exploring the subjective experience of professionals in the process of implementing aspects of OD in their practice as well as of taking part concurrently in the action-research, aiming to support the introduction and implementation of OD initially in the context of the Day Centre and later in the wider organization of E.P.A.P.S.Y. (Dawson et al., 2020).

Findings from the professionals’ focus group suggest that the implementation of OD has impacted mental health professionals across two main areas: their clinical practice and the group dynamics in the OD team.

Mental health professionals in this study expressed a difficulty in linking the theory with the practice of OD, especially with respect to implementing dialogical ways of being with others, particularly when working with service-users in crisis. The notion of reflective practice is regarded as crucial; however, professionals appear uncertain as to how to maintain appropriate boundaries between genuine, reflective practice and self-disclosure. Equally, maintaining a not-knowing stance is acknowledged as the greatest challenge for therapists, particularly under difficult circumstances where regressing to pre-existing psychiatric practices and notions of expertise relieve professional anxiety and restore a sense of control over the therapeutic process (Seikkula and Olson, 2003; Skourteli et al., 2019; Stylianidis, 2019b). Therapists in the present study report that containment of uncertainty was experienced as an absence of pressure to respond immediately to both network and their own expectations of themselves as omnipotent therapists, both during each meeting and overall, during the service user’s course of recovery. Sometimes the use of monological responses around critical issues of medical care and risk to self or others (as in cases of domestic violence) was deemed as necessary, however therapist attunement, flexibility and capacity to adjust to the ongoing network needs allowed them to gradually restore a dialogical stance (Borchers, 2014; Stockmann et al., 2017; Schubert et al., 2020). Although these challenges are most likely due to the lack of experience and formal, systematic training in OD, they are consistent with findings reported in the literature. According to Seikkula (2011), a significant portion of experienced and skilled mental health professionals present difficulties with the notion of dialogism since this is not a method or a technique but a way of being with others. In that respect, therapists who are required to participate in a meaningful, embodied and genuine way in the here-and-now, may often feel uncertain as to the experiential ways of implementing a dialogical stance (Seikkula and Arnkil, 2013; Buus et al., 2017, 2022; Ong and Buus, 2021; Kinane et al., 2022).

The notion of a cultural fit of Open Dialogue across different cultural and social contexts was acknowledged as an important parameter to be taken into account by participants in this study. Professionals seems to develop a less idealized view of Open Dialogue and to gain a more realistic view of what works for whom in psychotherapy (Norcross and Wampold, 2011). Participants report that the mentality and relationships among different members determine the quality and openness of the dialogue during network meetings. Further, the attitudes, culture and philosophy of each network seems crucial in the communication, sensitivity, and openness towards dialogical interventions; this is consistent with literature posing the issue of a realistic therapeutic and cultural match between approach and client (Johansen and Bille, 2005; Ong et al., 2019; Tribe et al., 2019). For example, Buus et al. (2017) report that families with a strong belief in authority and an expectation of being directed by mental health professionals may find the open format of the approach confusing and frustrating. Indeed, bearing in mind the Hellenic culture that values hierarchy and expertise, some families in the present study both expected and insisted on receiving direct advice and solutions from co-therapists and seemed to be lacking the capacity to contain the dialogical aspect of the interventions; for such networks, polyphony was viewed as chaotic, unhelpful and confusing thus preventing opportunities for observing small changes in the dynamics of the network over time. In cases where therapists resorted to more monological interventions, they report that it was their capacity to internally maintain a dialogical stance that allowed them to restore polyphony when the networks’ capacity to accommodate them was reinstated; this recommendation has also been made by Ong and Buus (2021). Professionals’ reflections from the focus group in the present study seem to suggest that therapists from different theoretical orientations utilized OD as a basis for integrating other aspects of psychotherapeutic practice according to individual networks’ needs (Seikkula and Arnkil, 2013; Buus et al., 2017; Dawson et al., 2019; Freeman et al., 2019).

Findings produced from the professionals’ focus group suggest that the introduction of Open Dialogue within the service continues to have a potent impact on group and organizational dynamics. Participants are reflecting and taking stock of the growing openness of the OD team over the past 5 years since the introduction of Open Dialogue in the service of the Day Centre. This openness essentially refers to the developing polyphony in the professionals’ team and within each participant separately, regarding new ideas, new people as well as several systemic changes within the organization. It also refers to an internal shift from a position of mistrust to a more open relational and philosophical stance towards self and others that may reflect the significant personal journey towards becoming a dialogical therapist. The experience of participating in the present research also appears to have changed over time; the professionals’ team seems to have moved away from fears of inadequacy and criticism to seeing the research as supportive of the implementation and as a valued opportunity for ongoing personal and professional development (Galbusera and Kyselo, 2019; Buus et al., 2022).

This process of becoming a dialogical therapist further seems to be reflected in the acknowledgement of boundaries and limitations of the professionals’ team, as produced by participant narratives. Therapists appear to be challenging the omnipotence and idealized view of team (as well as Open Dialogue approach itself) encountered in the early phases of the study and to be moving away from notions of monology, authority and expertise towards a position of greater internal and external polyphony.

Looking back, it appears as though the introduction of the Open Dialogue approach in this multidisciplinary team of mental health professionals has instigated a macroscopic transformative process in aspects of the organization itself. Firstly, it seems to have incited rapid changes in the constitution of the professionals’ team as well as a significant structural reform across different levels of management over time. Since such changes were often experienced as traumatic by employees, as reflected by references to the high turnover of staff over the past 5 years, the management of the organization introduced regular supervision (both clinical and group) in order to reduce conflict and promote tolerance and polyphony within the team, as informed by early findings of the study. It needs to be noted here that it was perhaps the lack of formal, systematic training in OD or other organizational characteristics prior and during the implementation process that may have contributed towards the overwhelming impact reported in participant narratives and not Open Dialogue as an approach *per se*. Indeed, over the course of the present action-research, there was ongoing dialogue, reflection and feedback between the research team, participants themselves and the management of the organization, in order to ensure that implementation attempts are guided and co-constructed through polyphony and co-operation across different levels. It appears that a greater investment is being made on the Open Dialogue approach over time through the acknowledgment of the pressing need for formal, systematic training as well as through attempts to expand the implementation of the Open Dialogue approach to other services of the organization (residential, mobile units, etc.), outside the Day Centre.

To sum up, the present action-research seems to have contributed significantly not only to the introduction and implementation the Open Dialogue approach within an established mental health service but also to the exploration of its impact upon professionals and organization with the view to supporting implementation attempts in the long-term. In short, the research presents a coherent story about the team’s journey with Open Dialogue over time; this journey may provide insight into the readiness of mental health professionals to adopt aspects of the Open Dialogue as well as the challenges and main issues that may emerge throughout this process.

## 5. Conclusions and limitations

A significant strength of the present implementation of Open Dialogue in Greece is that it has been developed in close collaboration with the two main Universities of Athens (Panteion University, Laboratory of Psychopathology, Social Psychiatry and Developmental Psychology and National and Kapodistrian University of Athens, Laboratory for Qualitative Research in Psychology and Psychosocial Well-being). The relationship to universities and academic departments has been recommended in the literature for the strengthening and institutionalizing of the Open Dialogue approach and for the development of larger research programs in the field of dialogical practices across different contexts (Buus et al., 2017).

The present paper highlights the pivotal role of mental health professionals in cultivating a new philosophy and practice in psychiatric care through presenting a multidisciplinary team’s journey with Open Dialogue and its transition from a monological to a dialogical epistemological stance. It seems important to highlight that even within innovative mental health organizations that are committed to the principles of recovery and empowerment, there are still significant collective defenses that may stem both from the threat to one’s professional identity and the deeply rooted impact of the paternalistic model in psychiatry (Hussain et al., 2018; Tribe et al., 2019; Stylianidis, 2019b).

In particular, the study may contribute towards the identification of the challenges and resistances encountered by mental health professionals with regard to issues of authority, hierarchy and expertise, when asked to engage in attempts that challenge notions of traditional psychiatric care. The findings emerging from the present study seem consistent with those reported in previous research (Buus et al., 2017; Ong and Buus, 2021; Kinane et al., 2022). Buus et al. (2017) report that the OD approach often generated resistance even amongst practitioners with formal training in OD, whose positions were challenged in different ways, although the authors remain skeptical as to whether such resistance is more pervasive compared to any approach that promotes reform of mental health services and includes the re-positioning of users and professional in the treatment setting; the authors go on to challenge the assumption of a universal ‘cultural’ fit between the OD approach and to acknowledge the characteristics of different networks (Buus et al., 2017). Similarly, Kinane et al. (2022) report that whilst for some service users, reflexive practice was experienced as strange and uncomfortable, professionals found the OD approach a valuable reflective space aiding the development of relationships and dialogue with each other and the acknowledgement of the power dynamics in the professionals’ team. Finally, Ong and Buus (2021) address the lack of precision and specificity around what constitutes dialogical practice that may contribute towards the ambiguity and uncertainty often encountered even by trained professionals. Overall, however, participants in the present study report experiencing Open Dialogue as enriching and valuable not only for their clinical practice but primarily for their personal development. Nevertheless, the present study further raises the question of the adaptability of the Open Dialogue approach across different contexts whilst highlighting the organizational parameters that are required for implementation attempts to be viable and sustainable over time. More research in the area certainly seems necessary to highlight challenges and issues encountered during implementation attempts of the model across different contexts.

However, the present study is not without limitations. Firstly, participants in the present study had not received any formal OD training and from that perspective the overall challenges and difficulties encountered may be due to the lack of exposure to experiential aspects of the model such as the use of the dialogical self. Furthermore, the present study included a very small sample of professionals, which may shed some light on a local level on one hand but may make generalization to other contexts somewhat difficult.

A crucial question that may remain is the notion of what works for whom in psychotherapy; as with other theoretical approaches the case may be that OD may be more or less compatible with some but not all service users and their networks, bearing in mind the clinical, cultural, educational and socio-economic variables of each network and setting. Within that, it seems important to safeguard the notion that the theoretical approach fits service-user needs rather than vice versa (Browne et al., 2019). Nevertheless, the perspective of consolidating and embracing Open Dialogue as a philosophical framework underpinning mental health care may further advance ongoing attempts towards psychiatric reform and a change of culture in psychiatric care with benefits on a micro, meso-and macro-levels of society.

## Data availability statement

The raw data supporting the conclusions of this article will be made available by the authors, without undue reservation.

## Ethics statement

The studies involving human participants were reviewed and approved by Association for Regional Development and Mental Health Ethics Committee National and Kapodistrian University of Athens Ethics Committee. The patients/participants provided their written informed consent to participate in this study.

## Author contributions

MS: organized, implemented and managed the action research, data analysis, writing up of the manuscript. PI: overall supervision of the research protocol and approved manuscript. LD, AA, GB, and AS: data collection and analysis, approved manuscript. SS: authorized implementation, overall supervision of the research protocol, and approved manuscript. All authors contributed to the article and approved the submitted version.

## Funding

Association for Regional Development and Mental Health (E.P.A.P.S.Y.) and the National and Kapodistrian University of Athens will be funding the present submission.

## Conflict of interest

The authors declare that the research was conducted in the absence of any commercial or financial relationships that could be construed as a potential conflict of interest.

## Publisher’s note

All claims expressed in this article are solely those of the authors and do not necessarily represent those of their affiliated organizations, or those of the publisher, the editors and the reviewers. Any product that may be evaluated in this article, or claim that may be made by its manufacturer, is not guaranteed or endorsed by the publisher.
